# Knowledge, attitudes, and practices regarding chronic sinusitis and its surgical treatment: a cross-sectional study in China

**DOI:** 10.7717/peerj.20633

**Published:** 2026-02-13

**Authors:** Maocai Li, Tan Wang, Lianqing Li, Lili Gong, Lingnan Bu

**Affiliations:** Department of Otorhinolaryngology Head and Neck Surgery, Liaocheng People’s Hospital, Liaocheng, China

**Keywords:** Knowledge attitude practice, Chronic sinusitis, Patients, Surgical treatment, Cross-sectional study

## Abstract

**Background:**

Chronic sinusitis is a common condition that can greatly affect patients’ quality of life. It is essential to understand patients’ knowledge, attitudes, and practices (KAP) regarding chronic sinusitis and its surgical treatment to enhance patient outcomes and optimize clinical management. This study aimed to investigate KAP among chronic sinusitis patients to identify areas for targeted intervention and improvement.

**Methods:**

A cross-sectional study was conducted with chronic sinusitis patients at the Ear, Nose, and Throat Department of Liaocheng People’s Hospital from June 2019 to June 2022. Demographic data and KAP were collected using self-designed questionnaires.

**Results:**

A total of 567 valid questionnaires were included, yielding a validity rate of 99.3%. Among the respondents, 314 were male (55.38%), and 260 individuals (45.86%) lived in urban areas. The mean scores for KAP were 7.86 ± 2.39 (possible range: 0–12), 17.69 ± 1.94 (possible range: 8–40), and 7.86 ± 2.39 (possible range: 7–35), respectively. Structural equation modeling demonstrated a direct impact of knowledge on attitude (β = −0.500, *p* < 0.001) and practice (β = 0.261, *p* = 0.001), and a direct impact of attitude on practice (β = 1.737, *p* < 0.001).

**Conclusion:**

Patients with chronic sinusitis demonstrated insufficient knowledge and negative attitudes and practices towards both the condition and its surgical treatment. These findings highlight the need for targeted interventions to improve patient education and awareness. By addressing knowledge gaps and fostering positive attitudes towards treatment options, healthcare providers can empower patients to make informed decisions and enhance their management of chronic sinusitis.

## Introduction

Chronic sinusitis is a common condition affecting 5–12% of the population and is associated with reduced quality of life, increased risk of lower respiratory tract diseases, and considerable healthcare costs (Kalińczak-Górna, Radajewski & Burduk, 2021; Tretiakow et al., 2022). When pharmacological treatments fail, surgical interventions such as endoscopic sinus surgery are often required (Kikuchi et al., 2021; Tretiakow et al., 2022). Despite its clinical importance, the effectiveness of treatment largely depends on patients’ understanding, beliefs, and behaviors.

Knowledge, Attitude, and Practice (KAP) surveys provide a structured way to assess patients’ awareness of disease, perceptions toward treatment, and adherence to medical recommendations (Nascimento et al., 2022; Rakotosamimanana et al., 2021). Prior studies in Western populations indicate that patients with chronic rhinosinusitis often lack adequate knowledge and may hold unrealistic expectations about surgical outcomes, which can undermine treatment satisfaction and compliance (Erskine et al., 2016; Mohamed & Abdel-Rehim, 2020). However, comprehensive KAP studies in Asian contexts, particularly in China, are limited.

To address this gap, we conducted a cross-sectional survey of chronic sinusitis patients in China to evaluate their knowledge, attitudes, and practices regarding both the disease and its surgical treatment.

## Materials and Methods

### Study design and participants

We conducted a cross-sectional study of patients with chronic sinusitis at the Department of Otorhinolaryngology, Liaocheng People’s Hospital, from June 2019 to June 2022. Ethical approval was obtained from the Ethics Committee of Liaocheng People’s Hospital [2019191], and informed consent was secured from all participants.

Inclusion criteria: (1) Patients diagnosed with chronic sinusitis according to the 2018 Chinese Diagnosis and Treatment Guidelines for Chronic Rhinosinusitis, with clinical symptoms and confirmation by nasal endoscopy and/or CT. (2) Age ≥18 years at the time of enrollment. (3) Have sufficient literacy and cognitive ability to understand and complete the questionnaire independently. Exclusion criteria: (1) Presence of acute sinusitis or other concurrent severe nasal diseases (*e.g*., nasal tumors, severe septal deviation). (2) History of sinus surgery prior to or during the study period.

### Questionnaire and quality control

The questionnaire design drew from relevant literatures (Mattos et al., 2020; Neubauer et al., 2016), the 2018 Chinese Diagnosis and Treatment Guidelines for Chronic Rhinosinusitis, and postoperative rehabilitation principles following endoscopic surgery for chronic sinusitis and nasal polyps (Liu et al., 2020). To ensure that the knowledge questions were appropriately challenging and comprehensible for the study population, we conducted a preliminary test with 33 participants, which provided feedback on question clarity and difficulty level. This initial testing phase allowed us to refine the questions, ensuring that they accurately reflected the participants’ understanding of chronic sinusitis. The preliminary test yielded a reliability coefficient of 0.852, supporting the internal consistency of the questionnaire.

The final questionnaire, in Chinese, covered four dimensions of information collection, comprising 38 items (Supplement Material). This included 10 items for basic information, 12 for the knowledge dimension, eight for the attitude dimension, and eight for the practice dimension. The knowledge dimension comprised 12 questions, scored 1 for correct and 0 for incorrect or unclear responses, with a score range of 0–12. The attitude dimension consisted of eight questions, using a five-point Likert scale ranging from very positive (five points) to very negative (one point), yielding a score range of 8–40. The practice dimension featured eight questions, with items 1–7 using a five-point Likert scale, scored based on the degree of positive behavior, ranging from always (five points) to never (one point), resulting in a score range of 7–35. Items lacking assignable scores were treated as a separate categorical variable. Achievement of scores exceeding 70% of the maximum in each section indicated sufficient knowledge, positive attitudes, and proactive practices (Lee & Suryohusodo, 2022).

The electronic questionnaire was generated using the Wen-Juan-Xing online platform (Changsha Ranxing Information Technology Co., Ltd; https://www.wjx.cn/app/survey.aspx), and a QR code was distributed to participants *via* Ding Talk. Participants were given the option to complete the survey either electronically or on paper. The online questionnaire could be accessed by scanning a QR code shared through the DingTalk platform, whereas printed copies were provided for in-person completion at the outpatient clinic. Respondents selected whichever format they found more convenient or familiar. During the process, medical staff refrained from offering health education or additional clarification to prevent bias in participants’ answers. To guarantee the reliability of the collected data, each IP address was limited to a single submission, all questions were compulsory, and the research team conducted thorough checks to confirm completeness, consistency, and overall validity.

### Sample size

Sample size was calculated using the formula for cross-sectional studies: α = 0.05, ${\rm \; n} = {\left( {\displaystyle{{{Z_{1 - \alpha /2}}} \over \delta }} \right)^2} \times p \times \left( {1 - p} \right)$ where ${Z_{1 - \alpha /2}}$ = 1.96 when α = 0.05, the assumed degree of variability of *p* = 0.5 maximises the required sample size, and δ is admissible error (which was 5% here). The theoretical sample size was 480 which includes an extra 20% to allow for subjects lost during the study.

### Statistical analysis

The analysis was performed using SPSS 22.0 (IBM Corp., Armonk, N.Y., USA) and AMOS 22.0 (IBM Corp., Armonk, N.Y., USA). Continuous variables were presented as mean ± standard deviation (SD), and comparisons between groups were conducted using *t*-tests or analysis of variance (ANOVA). Categorical variables were expressed as n(%). Univariate and multivariate linear regression analyses were utilized to identify factors associated with Practice. Structural Equation Modeling (SEM) confirmed direct impacts, where Knowledge exerted a direct effect on Attitude and Practice, and Attitude had a direct impact on Practice. The statistical tests were two-sided, and a *p*-value of less than 0.05 was considered statistically significant.

## Results

### Participant demographics

Initially, 571 questionnaires were collected. Among them, one completely repeated questionnaire, two questionnaires whose respondents were younger than 18 years old and one questionnaire with incomplete answers were excluded. Finally, a total of 567 valid questionnaires (valid rate: 99.3%) were included. Among the responders, 314 (55.38%) were males, and 260 (45.86%) resided in urban areas. Of the participants, 166 (29.28%) had been dealing with sinusitis for more than 10 years. However, in our cohort, only a relatively small proportion of surgical patients reported receiving preoperative systemic steroids, which may reflect variations in clinical practice compared with current guideline recommendations. The participants varied in terms of age, marital status, education level, occupation, and monthly income, which provided insights into potential demographic differences influencing KAP scores.

### Overall KAP scores

The mean knowledge, attitude, and practice scores were 7.86 ± 2.39 (possible range: 0–12), 17.69 ± 1.94 (possible range: 8–40), and 7.86 ± 2.39 (possible range: 7–35), respectively. These scores indicate a moderate level of knowledge and attitude, while practice scores suggest further room for improvement in adherence to sinusitis management practices. Demographic differences analyses indicated that, except for differences in treatment and medication, variations in all other collected demographic characteristics likely contributed to differences in participants’ knowledge, attitude, and practice scores (all *p* < 0.05) (Table 1).

**Table 1:** Demographic characteristics and KAP scores.

	*N* (%)	Knowledge		Attitude		Practice	
		Mean ± SD	*p*	Mean ± SD	*p*	Mean ± SD	*p*
**Total score**	567	7.86 ± 2.39		17.69 ± 1.94		7.86 ± 2.39	
**Gender**			<0.001		0.001		<0.001
Male	314 (55.38)	7.38 ± 2.41		17.45 ± 1.56		27.51 ± 4.90	
Female	253 (44.62)	8.44 ± 2.23		17.98 ± 2.31		31.17 ± 2.70	
**Age (years)**	43.86 ± 12.09						
**Residence**			<0.001		<0.001		<0.001
Rural	157 (27.69)	6.59 ± 1.93		17.91 ± 2.58		26.50 ± 3.14	
Urban	260 (45.86)	8.91 ± 2.33		18.37 ± 1.26		31.42 ± 1.76	
Suburban	150 (26.46)	7.36 ± 2.10		16.27 ± 1.33		27.95 ± 6.47	
**Education**			<0.001		0.001		<0.001
Junior High School and Below	195 (34.39)	6.57 ± 1.71		17.28 ± 2.56		25.58 ± 5.41	
High School/Technical School	173 (30.51)	7.83 ± 2.71		17.77 ± 1.23		30.67 ± 2.57	
College/Bachelor’s	199 (35.10)	9.14 ± 1.94		18.02 ± 1.67		31.29 ± 1.78	
**Occupation**			<0.001		<0.001		<0.001
Party leader of national government organizations, business, and public institutions	76 (13.40)	9.07 ± 1.02		18.33 ± 1.39		30.37 ± 1.07	
Professionals (Teachers, Doctors, Engineers, Writers, *etc*.)	96 (16.93)	8.76 ± 2.76		17.80 ± 1.77		33.02 ± 0.92	
Business and Service Personnel	81 (14.29)	7.81 ± 2.87		16.93 ± 0.93		31.05 ± 2.36	
Agricultural, Forestry, Animal Husbandry, Fisheries and Water Conservancy Workers	168 (29.63)	7.10 ± 1.80		17.70 ± 2.71		27.60 ± 3.31	
Production/Transport Equipment Operators	66 (11.64)	8.47 ± 2.66		18.12 ± 1.58		28.53 ± 2.75	
Other	80 (14.11)	6.75 ± 2.11		17.34 ± 1.31		25.11 ± 7.75	
**Monthly income (CNY)**			<0.001		0.021		<0.001
<5,000 (approximately USD 700)	205 (36.16)	6.63 ± 1.69		17.78 ± 2.53		28.60 ± 3.81	
5,000–10,000 (approximately USD 700–1,400)	252 (44.44)	8.38 ± 2.69		17.82 ± 1.57		28.35 ± 5.24	
10,000–20,000 (approximately USD 1,400–2,800)	110 (19.40)	8.95 ± 1.70		17.23 ± 1.32		31.97 ± 1.63	
**Marital status**			<0.001		<0.001		0.006
Unmarried	74 (13.05)	9.24 ± 1.86		16.97 ± 1.02		30.66 ± 3.61	
Married	449 (79.19)	7.68 ± 2.37		17.74 ± 2.08		28.95 ± 4.67	
Divorced/Widowed	44 (7.76)	7.32 ± 2.57		18.34 ± 1.22		28.52 ± 2.75	
**Health insurance**			<0.001		0.008		<0.001
Urban employee basic medical insurance	189 (33.33)	8.96 ± 2.13		18.06 ± 1.61		31.33 ± 2.84	
New rural cooperative medical insurance	313 (55.20)	7.14 ± 2.40		17.55 ± 2.07		27.38 ± 4.87	
Urban resident basic medical insurance	65 (11.46)	8.11 ± 1.67		17.26 ± 2.09		31.26 ± 1.53	
**Duration of chronic sinusitis**			<0.001		<0.001		0.001
≤3 years	127 (22.40)	8.24 ± 2.30		17.90 ± 1.75		29.24 ± 4.49	
(3–6) years	150 (26.46)	8.49 ± 2.12		18.05 ± 2.17		30.21 ± 3.12	
(6–10) years	124 (21.87)	7.03 ± 2.30		16.99 ± 1.32		29.05 ± 4.60	
>10 years	166 (29.28)	7.60 ± 2.54		17.72 ± 2.13		28.16 ± 5.12	
**Treatment and medication (Multiple choice)**							
Intranasal corticosteroid sprays (*e.g*., budesonide, fluticasone propionate, mometasone furoate)	314 (55.38)	8.87 ± 1.95		17.66 ± 1.58		30.42 ± 3.34	
Antihistamines and leukotriene antagonists (*e.g*., Loratadine, Cetirizine)	25 (4.41)	9.48 ± 1.33		17.84 ± 2.82		31.56 ± 1.16	
Mucolytics and decongestants (*e.g*., Eucalyptus oil capsules)	80 (14.11)	9.48 ± 2.17		17.74 ± 1.16		30.74 ± 3.23	
Traditional Chinese medicine/Herbal medicine	210 (37.04)	7.32 ± 2.19		17.82 ± 2.50		29.94 ± 3.64	
Nasal irrigation	70 (12.35)	10.03 ± 1.26		17.77 ± 1.67		30.26 ± 2.57	
Detoxification and analgesia	76 (13.40)	6.67 ± 1.89		17.97 ± 1.61		26.15 ± 6.44	
Others (Naproxen, seroganaphthalamine nasal spray, Dianjing, Biyuan Tongqiao granule, headache ning capsule, naphthalamine nasal spray, *etc*)	29 (5.11)	7.55 ± 2.57		17.97 ± 1.82		26.34 ± 4.34	
None	56 (9.88)	6.82 ± 2.57		17.18 ± 1.51		27.54 ± 4.42	

### Distribution of response to KAP dimensions

In the knowledge dimension, the two items with the highest correctness rates were “*The main symptoms of chronic sinusitis are nasal congestion, sticky or purulent nasal discharge”* (K1) with 96.83% and “*Sinusitis will not recur after surgery. (False)”* (K10) with 93.47%. The two items with the lowest correctness rates were “*The main purpose of surgery is to remove irreversible lesions in the nasal cavity and sinuses, reconstruct the ventilation and drainage of the nasal cavity and sinuses, promote the resolution of mucosal inflammation, and restore the function of mucosal glands and cilia”* (K6) with 12.87% and “*Surgery cannot remove or alter the inflammatory nature of the nasal sinus mucosa; continuous postoperative care and comprehensive medication are necessary to promote the gradual restoration of the morphology and function of the nasal sinus mucosa”* (K8) with 17.99%. This disparity in knowledge item scores highlights specific misconceptions about sinusitis surgery and postoperative care, suggesting a need for targeted patient education on these aspects (Table S1).

Patients exhibited diverse attitudes regarding sinusitis. A total of 53.44% wanted to know more about the disease (A1). A total of 78.66% were satisfied with the significant improvement in their lives due to surgery (A3). However, 70.37% and 85.71% expressed embarrassment (A4) and anxiety (A5) due to the disease, respectively. Additionally, over 95% expressed concern about sinusitis recurrence, which underscores the prevalent anxiety and psychological burden associated with the disease. Moreover, more than 95% were concerned about the recurrence of sinusitis, irrespective of whether they had undergone surgery or not (A6 and A7) (Table S2). Regarding the knowledge question about saline irrigation (K5: “Any concentration of saline irrigation can effectively improve symptoms”), the item was defined as false according to current evidence, which highlights the differential effects of hypertonic and isotonic saline solutions. While both concentrations may provide some symptom relief, research indicates varying efficacy levels depending on specific symptoms and patient profiles. We also recognize that patients might subjectively perceive relief with either concentration, which suggests that this item may have limited discriminatory power in reflecting patient knowledge and could be further refined in future surveys.

In terms of related practices, 74.43% of participants consistently adhered to the prescribed postoperative medication (P1). Additionally, 62.08% frequently paid attention to their emotions and sought professional help when needed (P5). However, despite the importance of regular health education, only 49.56% occasionally attended knowledge seminars, and 74.07% relied on self-directed learning through the Internet for relevant information (P7 and P8) (Table S3).

### Structural equation modeling

**Figure 1:**
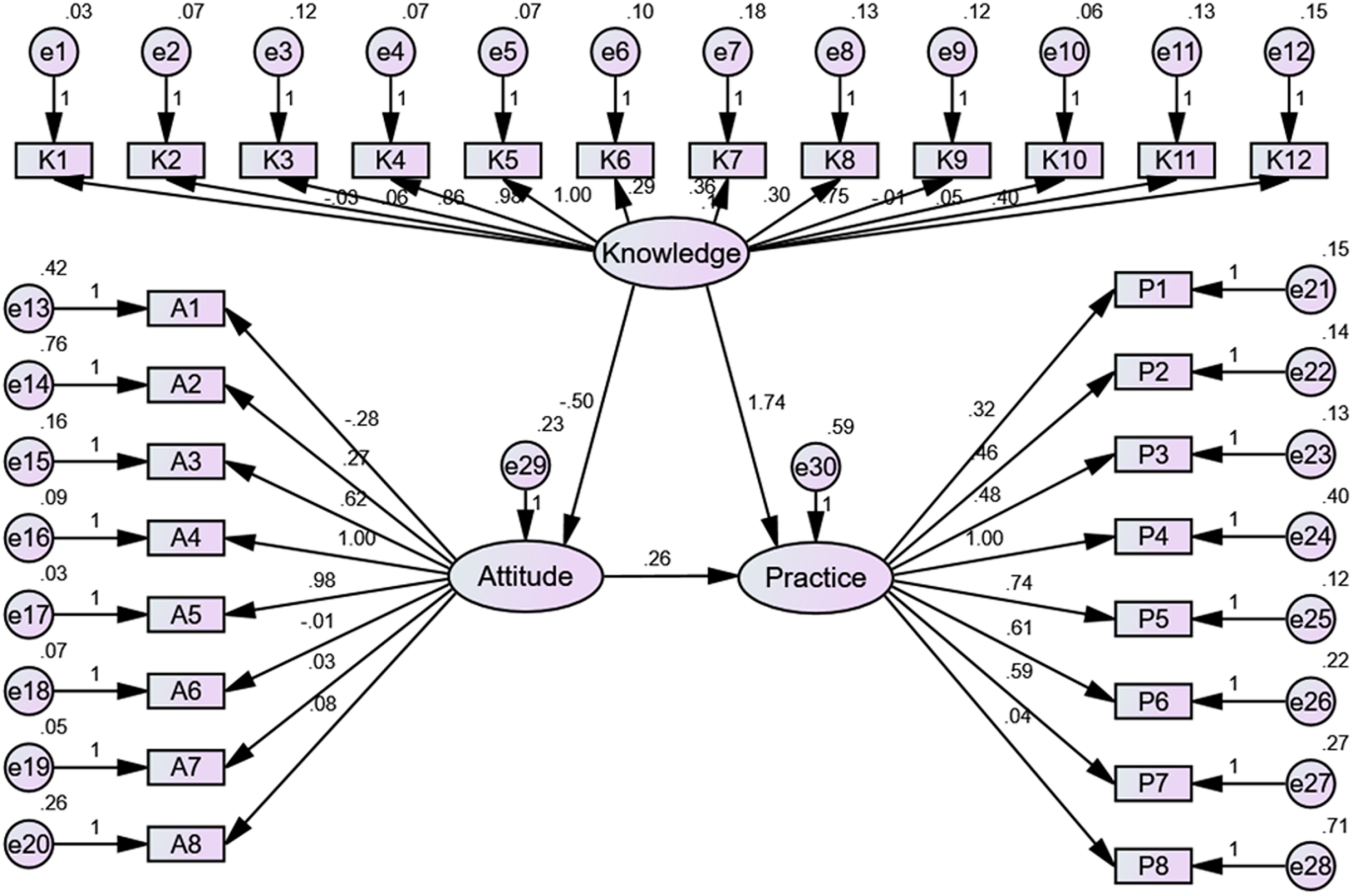
Structural equation modeling (SEM) results.

SEM indicated a direct impact of knowledge on attitude (β = −0.500, *p* < 0.001) and practice (β = 0.261, *p* = 0.001), and a direct impact of attitude on practice (β = 1.737, *p* < 0.001). This structural model illustrates the complex interplay among knowledge, attitudes, and practices, revealing that higher knowledge levels are associated with improved practices but may negatively influence attitudes, perhaps due to increased awareness of the chronic nature and recurrence risks of sinusitis (Fig. 1 and Table 2).

**Table 2:** SEM results.

			Estimate	*p*
Attitude	<—	Knowledge	−0.500	<0.001
Practice	<—	Attitude	1.737	<0.001
Practice	<—	Knowledge	0.261	0.001

### Multivariable analysis for practice

Multivariable linear regression analysis revealed that being male (β = −4.242, 95% CI [−5.044 to 3.440], *p* < 0.001), age (β = 0.093, 95% CI [0.055–0.130], *p* < 0.001), residing in urban areas (β = 8.153, 95% CI [6.999–9.307], *p* < 0.001), having a monthly income of 5,000–10,000 CNY (approximately USD 700–1,400) (β = −1.910, 95% CI [−2.941 to −0.878], *p* < 0.001), being married (β = −2.146, 95% CI [−3.406 to −0.877], *p* = 0.001), being divorced/widowed (β = −3.224, 95% CI [−5.187 to −1.260], *p* = 0.001), and having an disease duration of more than 3 years (*p* < 0.05) were independently associated with practice scores (Table 3).

**Table 3:** Multivariable linear regression analysis of factors associated with practice scores.

	β (95% CI)	*p*
**Gender**		
Male	−4.242 [−5.044 to −3.440]	<0.001
Female	Ref.	
**Age (years)**	0.093 [0.055–0.130]	<0.001
**Residence**		
Rural	Ref.	
Urban	8.153 [6.999–9.307]	<0.001
Suburban	0.006 [−1.167 to 1.179]	0.992
**Monthly income (CNY)**		
<5,000	Ref.	
5,000–10,000	−1.910 [−2.941 to −0.878]	<0.001
10,000–20,000	−0.283 [−1.5459 to 0.993]	0.664
**Marital status**		
Unmarried	Ref.	
Married	−2.146 [−3.406 to −0.877]	0.001
Divorced/Widowed	−3.224 [−5.187 to −1.260]	0.001
**Duration of chronic sinusitis**		
≤3 years	Ref.	
(3–6) years	1.586 [0.472–2.700]	0.005
(6–10) years	−1.559 [−2.766 to −0.351]	0.012
>10 years	−1.399 [−2.595 to −0.202]	0.022

## Discussion

This study reveals that patients with chronic sinusitis exhibit insufficient knowledge, negative attitudes, and suboptimal practices regarding the condition and its surgical treatment. To improve clinical care for these individuals, it is recommended to implement targeted educational initiatives addressing knowledge gaps. Educational efforts should be customized based on demographic factors, leveraging tailored communication strategies and actively dispelling common misconceptions. Furthermore, interventions aimed at fostering positive attitudes and enhancing knowledge have the potential to positively impact healthcare practices.

Our analysis of KAP in chronic sinusitis patients uncovered significant variations across demographic and health-related parameters. A noteworthy finding is the gender disparity, with males displaying significantly lower knowledge scores than females. This aligns with existing literature on gender-specific variations in health-related knowledge levels, underscoring the necessity for targeted interventions tailored to each gender group (Zheng et al., 2021). Residential location also emerged as a pivotal factor influencing KAP scores, with urban residents consistently outperforming their rural and suburban counterparts. This finding reflects similar studies on health literacy, emphasizing an urban-rural gap requiring attention in public health interventions (Wang et al., 2021; Wu, Kc & Luy, 2022). The positive correlation between educational attainment and KAP scores aligns with established literature, emphasizing the role of education in shaping health-related knowledge, attitudes, and practices (Chen et al., 2020). Incomes also exhibited a significant association, with higher income levels correlating with superior KAP scores. This socioeconomic gradient in health knowledge and practices underscores the need for strategies addressing economic disparities, such as cost-effective educational campaigns and subsidized healthcare initiatives (Poddighe & Vangelista, 2020; Zhou et al., 2020). The multivariable analysis identified several significant demographic and clinical factors influencing practice scores. Male patients exhibited markedly lower practice scores, consistent with previous literature documenting gender disparities in healthcare compliance behaviors. Age demonstrated a positive correlation with practice scores, suggesting that older patients may be more diligent in following treatment protocols, possibly due to accumulated experience in managing chronic conditions and greater health consciousness. Urban residence strongly predicted higher practice scores, reinforcing our earlier discussion of urban-rural healthcare disparities. Interestingly, middle-income patients showed lower practice scores compared to lower-income groups, possibly reflecting competing time demands and work-related stress among this demographic. Marital status emerged as another significant predictor, with both married and divorced/widowed individuals showing lower practice scores compared to unmarried participants, challenging conventional assumptions about marital support enhancing healthcare adherence. Disease duration also showed varying effects on practice scores, indicating that prolonged experience with chronic sinusitis might influence treatment adherence patterns in complex ways that warrant further investigation.

SEM results indicating a negative impact of knowledge on attitudes in chronic sinusitis patients are somewhat surprising. One possible explanation could be that individuals with elevated knowledge levels in chronic sinusitis may adopt a more critical stance, fostering a cautious or skeptical attitude, particularly toward medical interventions (Lewis & Holm, 2022). This negative impact may reflect underlying apprehensions about surgical treatments, as patients may have reservations regarding procedural complexities, recovery challenges, or potential complications—aligning with previous research that highlights patient concerns surrounding surgical interventions (Cheng et al., 2021; Shi et al., 2022).

The study underscores significant gaps in knowledge among individuals with chronic sinusitis, particularly in recognizing symptoms, understanding medication, and postoperative care. The relatively low rate of preoperative steroid use among surgical patients observed in this study may be explained by variations in clinical practice, limited adherence to guideline recommendations in some hospitals, and patients’ concerns about potential steroid side effects. This discrepancy suggests the need for reinforcing guideline-based education for both healthcare providers and patients. Misunderstandings about surgery as a standalone solution pose risks to long-term outcomes, particularly when attitudinal barriers, such as underestimating the need for continued care, complicate postoperative management. Targeted educational efforts are essential, focusing on clarifying symptoms, emphasizing the importance of glucocorticoids, and dispelling misconceptions surrounding surgery’s overall effectiveness. Patient attitudes—such as the belief that surgery is a permanent solution without the need for ongoing care—challenge adherence to postoperative protocols. Practices also indicate a need for targeted interventions, with varying compliance in areas like quitting smoking and regular check-ups. A considerable proportion of patients neglect to prioritize emotional well-being, underscoring the importance of integrating mental health support within postoperative care plans. To address these identified weaknesses, comprehensive educational interventions are recommended. Patient counseling sessions, accessible educational materials, and interactive workshops—guided by health literacy principles and tailored to chronic sinusitis patients—are essential for enhancing patient knowledge and engagement (Mbanda et al., 2021; Melnic et al., 2022; Murugesu et al., 2022). Additionally, efforts should be directed towards addressing attitudes hindering postoperative care adherence, emphasizing the necessity of ongoing medical supervision and holistic self-care practices. Patient-centered care models, which incorporate shared decision-making, have demonstrated success in improving adherence and patient outcomes, suggesting their applicability to this patient population (Hermans et al., 2022; Mapes, DePergola & McGee, 2020). Additionally, it is important to consider the differences in KAP among chronic sinusitis patients opting for conservative, non-surgical treatments. Patients who choose conservative management may have varying levels of knowledge, distinct attitudes towards medical interventions, and different self-care practices compared to those opting for surgical treatment. These differences could impact their adherence to recommended treatments and influence their overall quality of life.

This study has several limitations that should be considered when interpreting the results. Firstly, the cross-sectional design restricts our ability to establish causality between variables and precludes dynamic analysis of patient data. Specifically, we could neither assess changes in patients’ knowledge levels before and after events such as nasal polyp recurrence, nor were we able to conduct an analysis of the relative weight of each individual KAP metric in relation to disease progression or outcomes. Longitudinal studies are therefore needed to provide a more robust understanding of these dynamic and differential relationships. Secondly, relying on self-reported data introduces the possibility of response bias and may not fully capture the nuances of participants’ experiences. Thirdly, and perhaps most significantly, the single-center focus of the study at Liaocheng People’s Hospital substantially limits the generalizability of our findings to the broader chronic sinusitis population in China and globally. Regional variations in healthcare access, educational systems, and cultural attitudes toward medicine likely influence patient KAP scores in ways our study cannot capture. Future research should incorporate multi-center designs across different regions of China and possibly international collaborations to provide more comprehensive and generalizable insights into chronic sinusitis KAP patterns. In conclusion, patients with chronic sinusitis demonstrated inadequate knowledge, negative attitudes and practices towards chronic sinusitis and its surgical treatment. To enhance clinical practice in managing chronic sinusitis, targeted interventions are warranted. Initiatives should concentrate on educational programs aimed at improving patient awareness and understanding of the condition, emphasizing the benefits of surgical treatment, and addressing prevalent misconceptions. Additionally, healthcare providers should tailor their communication strategies based on identified demographic factors such as gender, age, urban residence, income, marital status, and disease duration.

## Supplemental Information

10.7717/peerj.20633/supp-1 Supplemental Information 1 Raw data.

10.7717/peerj.20633/supp-2 Supplemental Information 2 Codebook.

10.7717/peerj.20633/supp-3 Supplemental Information 3 Supplementary Tables.

10.7717/peerj.20633/supp-4 Supplemental Information 4 Questionaire.

10.7717/peerj.20633/supp-5 Supplemental Information 5 STROBE checklist.

## Additional Information and Declarations

### Data Availability

The following information was supplied regarding data availability:

The raw data are available in the Supplemental File.
