# Supplementary material for: Knowledge, attitudes, and practices regarding chronic sinusitis and its surgical treatment: a cross-sectional study in China
Source: PeerJ. 2026 Feb 13;14:e20633. doi: 10.7717/peerj.20633 (PMC12908572; doi:10.7717/peerj.20633)
Supplement: Supplemental Information 2 [file peerj-14-20633-s002.docx]

| **Gender** |
| --- |
| a. Male |
| b. Female |
| **Residence** |
| 1. Rural |
| 1. Urban |
| 1. Suburban |
| **Education** |
| 1. Junior High School and Below |
| 1. High School/Technical School |
| 1. College 2. Bachelor’s and above |
| **Occupation** |
| 1. Party leader of national government organizations, business, and public institutions |
| 1. Professionals (Teachers, Doctors, Engineers, Writers, etc.) |
| 1. Business and Service Personnel |
| 1. Agricultural, Forestry, Animal Husbandry, Fisheries and Water Conservancy Workers |
| 1. Production/Transport Equipment Operators 2. Office staff and related personnel 3. Armyman 4. Others |
| **Monthly Income (CNY)** |
| 1. <2000 |
| 1. 2000-5000 |
| 1. 5000-10000 2. 10000-20000 |
| **Marital Status** |
| 1. Unmarried |
| 1. Married |
| 1. Divorced 2. Widowed |
| **Health Insurance** |
| 1. Urban Employee Basic Medical Insurance |
| 1. New Rural Cooperative Medical Insurance |
| 1. Urban Resident Basic Medical Insurance 2. Retired Cadre Medical Insurance |
| K1-K12   1. Correct 2. Wrong 3. Unclear   A1-A8  a. Strongly agree  b. Agree  c. Unsure  d. Disagree  e. Strongly disagree |
| P1-P7   1. Always 2. Often 3. Sometimes 4. Rarely 5. Never   P8   1. Medical education 2. Self-research on the Internet 3. Hospital/Department disseminated popular science articles 4. Newspapers/magazines/TV/radio 5. Other |
