# Supplementary material for: Knowledge, attitudes, and practices regarding chronic sinusitis and its surgical treatment: a cross-sectional study in China
Source: PeerJ. 2026 Feb 13;14:e20633. doi: 10.7717/peerj.20633 (PMC12908572; doi:10.7717/peerj.20633)
Supplement: Supplemental Information 4 [file peerj-14-20633-s004.docx]

**Knowledge, Attitude, and Practices Regarding Chronic Sinusitis and Surgical Treatment among Patients**

**Part I Basic Information**

| **Gender** |
| --- |
| Male |
| Female |
| **Age (years)** |
| **Residence** |
| Rural |
| Urban |
| Suburban |
| **Education** |
| Junior High School and Below |
| High School/Technical School |
| College/Bachelor’s |
| **Occupation** |
| Party leader of national government organizations, business, and public institutions |
| Professionals (Teachers, Doctors, Engineers, Writers, etc.) |
| Business and Service Personnel |
| Agricultural, Forestry, Animal Husbandry, Fisheries and Water Conservancy Workers |
| Production/Transport Equipment Operators |
| Other |
| **Monthly Income (CNY)** |
| <5000 |
| 5000-10000 |
| 10000-20000 |
| **Marital Status** |
| Unmarried |
| Married |
| Divorced/Widowed |
| **Health Insurance** |
| Urban Employee Basic Medical Insurance |
| New Rural Cooperative Medical Insurance |
| Urban Resident Basic Medical Insurance |
| **Duration of** **chronic sinusitis** |
| ≤3 years |
| (3-6] years |
| (6-10] years |
| ＞10 years |
| **Treatment and Medication (Multiple choice)** |
| Hormonal Nasal Sprays (e.g., Budesonide Nasal Spray, Fluticasone Propionate Nasal Spray, Mometasone Furoate Nasal Spray) |
| Antihistamines and Leukotriene Antagonists (e.g., Loratadine, Cetirizine) |
| Mucolytics and Decongestants (e.g., Eucalyptus Oil Capsules) |
| Traditional Chinese Medicine/Herbal Medicine |
| Nasal Irrigation |
| Detoxification and Analgesia |
| Others (Naproxen, seroganaphthalamine nasal spray, Dianjing, Biyuan Tongqiao granule, headache ning capsule, naphthalamine nasal spray, etc) |
| None |

**Part Ⅱ Knowledge**

| 1. The main symptoms of chronic sinusitis are nasal congestion, sticky or purulent nasal discharge. | Correct | Wrong | Unclear |
| --- | --- | --- | --- |
| 2. Chronic sinusitis does not present with headache, facial pain, decreased or loss of smell. (False) | Correct | Wrong | Unclear |
| 3. Glucocorticoids have significant anti-inflammatory, anti-edema, and immunosuppressive effects. They are the most important drugs in the treatment system, administered systemically (oral) or locally (nasal). | Correct | Wrong | Unclear |
| 4. Nasal saline irrigation can improve symptoms and quality of life, protecting the nasal sinus mucosa. | Correct | Wrong | Unclear |
| 5. Any concentration of saline irrigation can effectively improve symptoms. (False) | Correct | Wrong | Unclear |
| 6. The main purpose of surgery is to remove irreversible lesions in the nasal cavity and sinuses, reconstruct the ventilation and drainage of the nasal cavity and sinuses, promote the resolution of mucosal inflammation, and restore the function of mucosal glands and cilia. | Correct | Wrong | Unclear |
| 7. After surgery, there is no need to continue regular medication; medication can be taken only when uncomfortable. (False) | Correct | Wrong | Unclear |
| 8. Surgery cannot remove or alter the inflammatory nature of the nasal sinus mucosa; continuous postoperative care and comprehensive medication are necessary to promote the gradual restoration of the morphology and function of the nasal sinus mucosa. | Correct | Wrong | Unclear |
| 9. Early postoperative nasal saline lavage is effective in removing nasal crusts and preventing adhesions. | Correct | Wrong | Unclear |
| 10. Sinusitis will not recur after surgery. (False) | Correct | Wrong | Unclear |
| 11. There are no dietary restrictions after sinusitis surgery; consumption of spicy, irritating, or seafood does not matter. (False) | Correct | Wrong | Unclear |
| 12. Early postoperative sneezing and coughing are acceptable. (False) | Correct | Wrong | Unclear |

| **Part III Attitude** | | | | | |
| --- | --- | --- | --- | --- | --- |
| 1. I want to learn more about the disease. | a. Strongly agree | b. Agree | c. Unsure | d. Disagree | e. Strongly disagree |
| 2. I believe surgery is a permanent solution, and I don't need to worry about it afterward. | a. Strongly agree | b. Agree | c. Unsure | d. Disagree | e. Strongly disagree |
| 3. Surgery has greatly improved my quality of life. | a. Surgical efficacy | b. Surgical cost | c. Surgical time | d. Experience of surgeon | |
| 4. Being sick makes me feel embarrassed. | a. Strongly agree | b. Agree | c. Unsure | d. Disagree | e. Strongly disagree |
| 5. Being sick makes me very anxious. | a. Strongly agree | b. Agree | c. Unsure | d. Disagree | e. Strongly disagree |
| 6. I am worried that the disease will recur even after surgery. | a. Strongly agree | b. Agree | c. Unsure | d. Disagree | e. Strongly disagree |
| 7. I am very afraid of a recurrence. | a. Strongly agree | b. Agree | c. Unsure | d. Disagree | e. Strongly disagree |
| 8. If someone around me is also troubled by sinusitis, I would recommend them to consider surgery. | a. Strongly agree | b. Agree | c. Unsure | d. Disagree | e. Strongly disagree |

| **Part IV Practice** |
| --- |

| 1. I can follow the doctor's advice and take medication regularly after surgery. | Always | Often | Sometimes | Rarely | Never |
| --- | --- | --- | --- | --- | --- |
| 2. I can undergo regular check-ups. | Always | Often | Sometimes | Rarely | Never |
| 3. I can adhere to nasal saline irrigation after discharge. | Always | Often | Sometimes | Rarely | Never |
| 4. I can quit smoking and drinking. | Always | Often | Sometimes | Rarely | Never |
| 5. I can maintain postoperative hygiene. | Always | Often | Sometimes | Rarely | Never |
| 6. I can prioritize my emotions and seek professional help when necessary. | Always | Often | Sometimes | Rarely | Never |
| 7. If there are knowledge lectures, I am willing to attend. |  |  |  |  |  |
| 8. Ways you acquire relevant knowledge |  |  |  |  |  |
| Medical education |  |  | | | |
| Self-research on the Internet |  |  | | | |
| Hospital/Department disseminated popular science articles |  |  | | | |
| Newspapers/magazines/TV/radio |  |  | | | |
| Other |  |  | | | |
